# Maternal and neonatal outcomes in Gaza amid armed hostilities in 2025

**DOI:** 10.1002/ijgo.70984

**Published:** 2026-03-30

**Authors:** Shaymaa Abuhaiba, Younis Elijla, Aya El‐Dadah, Othman Nassar, Meran Abu‐Sultan, Maysaa Almasry, Hebatallah Alkhatib, Mahmoud Alsady, Amir Mahdi Ghafarian, Muath Alsarafandi, Lina Abumughessib, Arham Ali, Yassar Arain, Abeerah Muhammad, Belal Aldabbour, Shayan Ali, Bilal Irfan

**Affiliations:** ^1^ Al‐Shifa Medical Complex Gaza Palestine; ^2^ Al‐Helou Maternity Hospital North Gaza Palestine; ^3^ Faculty of Medicine, Islamic University of Gaza Gaza Palestine; ^4^ College of Medicine International University of the Health Sciences Basseterre Saint Kitts and Nevis; ^5^ Al‐Aqsa Martyrs' Hospital Deir Al‐Balah Palestine; ^6^ Division of Pediatric Critical Care, Department of Pediatrics Loma Linda University Children's Hospital Loma Linda California USA; ^7^ Department of Pediatrics Texas Christian University School of Medicine Fort Worth Texas USA; ^8^ Baylor Scott and White Health Dallas Texas USA; ^9^ Neuroscience Department Austin College Sherman Texas USA; ^10^ North Texas Medical Research Institute Rockwall Texas USA; ^11^ Center for Bioethics Harvard Medical School Boston Massachusetts USA; ^12^ Center for Surgery and Public Health Brigham and Women's Hospital Boston Massachusetts USA; ^13^ Department of Neurology University of Michigan Medical School Ann Arbor Michigan USA; ^14^ Department of Epidemiology University of Michigan School of Public Health Ann Arbor Michigan USA

**Keywords:** anemia in pregnancy, armed conflict, facility‐based observational study, food insecurity, forced displacement, Gaza, maternal health, neonatal outcomes, Palestine

## Abstract

**Objective:**

This study documents facility‐based maternal, obstetric, and neonatal outcomes and associated conflict‐related exposures and living conditions among pregnancy‐related encounters at Al‐Helou Maternity Hospital in Gaza from late April to early September 2025, with a small number of additional encounters recorded at Al‐Shifa Medical Complex.

**Methods:**

We conducted an observational, facility‐based study using a combined retrospective‐prospective approach. All recorded obstetric encounters were eligible. Outpatient antenatal clinic visits were excluded. Data were abstracted from routine clinical records and contemporaneous intake forms/clinician notes. Analyses were primarily descriptive (medians [interquartile range, IQR] and counts/percentages with variable‐specific denominators). Exploratory correlation analyses examined birthweight in relation to selected maternal exposures.

**Results:**

Among 686 women (median age 28 years [IQR 23–33]), 92.9% reported ≤2 meals/day and 71.7% had anemia (Hb <11 g/dL); 78.7% were currently displaced (54.8% living in tents), 57.0% reported nearby bombing within 24 h, 98.0% had smoke exposure, and 74.6% reported heavy lifting. Only 32.1% reported access to prenatal care, 52.6% pregnancies were unplanned, and 11.9% of unplanned pregnancies reported successfully accessing contraception. Gestational age was ≥37 weeks in 80.8%. Delivery management included 57.4% normal vaginal deliveries and 37.6% caesarean sections (53.4% due to prior caesarean). Postpartum intensive care unit admission occurred in 1.0% with 28.6% mortality among those admitted. Neonatal outcomes (*n =* 665) included median birthweight 3000 g (IQR 2600–3300), neonatal intensive care unit (NICU) admission 12.9%, discharge alive 98.6%, and last documented Apgar score of 10 in 43.0% (0 in 9.5%).

**Conclusion:**

Pregnancy‐related encounters in Gaza occurred amid pervasive food scarcity, displacement, smoke exposure, and recent bombardment, alongside high anemia prevalence and limited prenatal care. Maternal care featured substantial caesarean use and severe maternal complications, while neonatal outcomes included frequent NICU admission, underscoring a high‐risk environment for mothers and newborns.

## 1 INTRODUCTION

Maternal health can often serve as an important barometer of a population's general health and the operational capacity of its baseline health services.^1^ Prior to October 2023, while the Gaza strip maintained a relatively functioning maternal healthcare system, it was inherently fragile due to a long‐standing siege and blockade imposed by Israel.^2^ Evidence of this pre‐existing vulnerability includes prevalence of anemia during pregnancy, which ranged from 35.7% to 42.8%, and increasing low birthweight prevalence in Gaza exceeding that of the occupied West Bank.^3^, ^4^, ^5^

The escalation of armed hostilities since 2023 has transformed what was a climate of chronic resource scarcity into an acute health crisis for expectant mothers and neonates through the systematic destruction of health care and civilian infrastructure and restrictions on food aid.^6^ In the first 6 weeks of the Israeli military campaign alone, 60.8% of health facilities sustained damage and 35.1% were destroyed and thereby put out of service.^7^ Further, highly destructive 2000‐lb bombs were detonated in dangerous proximity to hospitals, with 83.3% of all hospitals falling within 800 m of the facility, resulting in widespread infrastructure damage and injuries.^8^ This magnitude of destruction, alongside the widespread denial of entry of food, fuel, and essential medical supplies (including baby formula) has resulted in fatal obstetric and neonatal outcomes and consequences.^9^, ^10^, ^11^ Israeli forces have repeatedly attacked neonatal intensive care unit (NICU) incubators and ventilation equipment, and denied fuel and electricity resupply attempts.^5^, ^12^, ^13^, ^14^, ^15^ This, coupled with the forced evacuation of medical staff and patients, has resulted in the death of newborns within hospitals and pregnant women, with scenes of babies decomposing within wards.^12^, ^13^, ^14^

As a consequence, maternal malnutrition and anemia have reached crisis levels, with estimates suggesting approximately 45% of pregnant women in Gaza to be suffering from malnutrition, with studied cohorts reporting a prevalence reaching 50.4%.^3^, ^16^ This nutritional depletion can contribute directly to below average fetal weights, increasing the risk of spontaneous abortion, stillbirth, and premature birth.^17^, ^18^ For the mother, severe anemia heightens the danger of life‐threatening complications like postpartum hemorrhage, with one United Nations (UN) estimate suggesting that 180 Palestinian women were giving birth daily amid the genocide.^9^, ^19^, ^20^

Simultaneously, chronic psychological stress stemming from constant aerial bombardment and numerous forced displacement events can exacerbate adverse outcomes.^21^ Surveys indicate that 90.42% of pregnant women reported moderate to high stress levels, with clinicians reporting that many women have given birth prematurely as a consequence of a constant state of terror and anxiety induced from Israel's military assault.^3^, ^10^, ^19^, ^22^, ^23^ This acute trauma is also driving a surge in stress‐related miscarriages and stillbirths as well as and preterm delivery.^24^, ^25^ Further, the unsanitary conditions in overcrowded shelters, coupled with exposure to air particles filled with pollution and smoke due to the extensive bombing campaign, can result in increases in infection and miscarriages.^26^, ^27^

While international bodies and aid agencies have provided macro‐level accounts of the severity of maternal and neonatal outcomes in Gaza, facility‐based primary clinical evidence has lagged behind. Destruction of the central server for health data collection in November 2023, as well as burning of hospital records, has impeded robust quantification of this conflict's impact on maternal health on a granular level.^28^, ^29^, ^30^, ^31^, ^32^, ^33^ This study aims to address this critical gap in the literature by documenting obstetric encounters at the Al‐Helou Maternity Hospital and Al‐Shifa Medical Complex, providing a comprehensive clinical account of reproductive health outcomes during conditions widely identified by human rights organizations and UN bodies as constituting genocide and the crime against humanity of extermination.^34^, ^35^, ^36^, ^37^, ^38^, ^39^, ^40^, ^41^, ^42^

## 2 METHODS

### 2.1 Study design and setting

We conducted an observational, facility‐based study using a combined retrospective‐prospective approach. We described maternal, obstetric, and neonatal outcomes from late April 2025 to early September 2025 after Israel's resumed large‐scale attacks on the Gaza Strip after violating the January 19 ceasefire agreement in March 2025. Data was retrospectively collected from hospital records and prospectively collected during the study period as well in the summer of 2025. The primary site of the study was at the Al‐Helou Maternity Hospital in the Gaza governorate, with a small number of patients also recorded from the nearby Al‐Shifa Medical Complex using the same data structure and variable definitions. The primary unit and point of analysis was the obstetric encounter by clinical staff (i.e., a pregnancy‐related presentation resulting in delivery, pregnancy loss management, or admission for obstetric care).

This study received ethical approval from the Palestinian Ministry of Health (Ref #: 2589122) and Al‐Helou Maternity Hospital and was conducted with respect to the guidelines set forth in the Declaration of Helsinki for active settings of armed conflict using clinical documentation and patient‐reported exposure information that was recorded during routine care under relevant informed consent guidelines. Analyses were performed on a dataset that was structured for documenting clinical outcomes, with reporting being in aggregate to reduce identifiability. No individual‐level identifying information is reported in this manuscript.

### 2.2 Participants and eligibility criteria

All obstetric encounters that were recorded in the study dataset were eligible for inclusion. Outpatient interactions at antenatal clinics were excluded. Encounters included deliveries (vaginal or caesarean), care provided for loss of pregnancy (including miscarriages and intrauterine fetal death), and ectopic pregnancy management. Multiple gestations were also included. For neonatal analysis, each fetus/newborn outcome that corresponded to a specific encounter was counted, such that the totals could exceed the number of maternal encounters.

### 2.3 Data sources and data collection procedures

Data were assembled from routine processes of documentation for maternity‐related cases. Demographic variables and those related to obstetric history (e.g., age, gravidity, parity/term, prior pregnancies, and living children) and pregnancy management variables (e.g., mode of delivery, and interventions) were taken from clinical records at the time of care or retrospectively from hospital when needed.

Conflict‐related exposures and living conditions were recorded as clinical encounters took place and patient history was documented through intake forms and/or contemporaneous clinician notes. These included the current displacement status, number of displacement events, type of residence, reported nearby bombing, and forced displacement events in the last 24 h, exposure to smoke, lifting heavy weights, prior and current‐pregnancy conflict‐related injury/trauma, and certain impacts on family (e.g., bereavement and spousal injury or disability). Neonatal variables such as sex, birthweight, Apgar scores, NICU admission, and the status at discharge were taken from delivery notes and documentation on newborns.

### 2.4 Measures

Maternal age was recorded in years. Obstetric history included gravidity and outcomes of prior pregnancies (term births, preterm births, and abortions) and the number of living children. Hemoglobin values were recorded based on the most proximal documented hemoglobin value. Medical comorbidities were recorded and extracted from the patients directly or from notes on past medical history. Pregnancy‐related characteristics were recorded, including gestational age at the time of presentation; prenatal care (recorded as any prenatal care reported); intention of pregnancy (planned vs. unplanned); and, for unplanned pregnancies, attempts to obtain contraception and, if so, the reported success in accessing such contraception and the availability of the contraceptive method after gaining access to it.

The management of current pregnancy was categorized as normal vaginal delivery, caesarean section, dilation and curettage, or other specified management protocols. For caesarean section, the documented primary indication was recorded when available and thereby categorized (e.g., previous caesarean section, breech, fetal distress, twins, other, or not identified). Additional intrapartum/postpartum variables included the induction of labor, assisted vaginal delivery, and postpartum intensive care unit (ICU) admission and indication, as well as the status of a patient following ICU admission.

Variables related to the exposure of conflict‐related elements were also recorded, primarily as binaries (yes/no) or count measures when applicable. These included a conflict‐related injury during or prior to the current pregnancy, disability resulting from a conflict‐related injury, a bombing event in the past 24 h (self‐report of a nearby bombing), a forced displacement in the past 24 h, current status of displacement, the number of displacement events since the start of the genocide, traumatic events during the current pregnancy, surgical intervention history for any conflict‐induced traumatic injury, living conditions (tent or not), exposure to smoke (self‐report of exposure to smoke from fires/combustion), lifting heavy objects (self‐report of lifting heavy objects during the current pregnancy), death of a child, death of a spouse due to conflict‐related injury, spouse's exposure to conflict‐related trauma, and any disability reported in a spouse as a consequence of the armed conflict.

The newborn and fetus dataset included recorded outcomes that corresponded to maternal encounters, including multiple gestations and pregnancy losses. Sex was recorded. Birthweight (in grams) was recorded for liveborn infants. Only the last Apgar score was recorded due to potential inaccuracies or lack of documentation of the time at which each of the Apgar scores were taken, and NICU admission was treated as a binary variable (yes/no). The status of the newborn at the time of discharge was also categorized (alive/dead), with the “dead” category in this dataset including abortions, ectopic pregnancies, intrauterine fetal deaths, and early neonatal deaths.

### 2.5 Operational definitions

Key definitions used for the analysis were as follows:

- Anemia in pregnancy: hemoglobin <11 g/dL (vs ≥11 g/dL)
- Gestational age categories: ≥37 weeks; 34–36; 32–33; 28–31; 24–27; and < 23 weeks
- Preterm birth: gestational age < 37 weeks
- Hypertensive disorders of pregnancy: Categorized as gestational hypertension, pre‐eclampsia, and eclampsia as documented clinically; premature rupture of membrane, placenta abruptio, placenta previa, post‐dates pregnancy, gestational diabetes mellitus, intrauterine growth restriction: recorded based on clinician documentation/diagnosis in the record
- Meals per day: Categorized as ≤2 vs. ≥3 meals/day based on patient report.

### 2.6 Statistical analysis

Analyses in this study were primarily descriptive. Continuous variables were summarized using medians with interquartile ranges (IQRs) and ranges wherever available. Categorical variables were summarized using counts and percentages with denominators reflecting the number of encounters with recorded data for the given variable.

To explore linear associations between newborn birthweight and selected exposure measures, we conducted correlation analyses reporting correlation coefficients and corresponding *P*‐values for the number of meals per day, category of hemoglobin level, planned pregnancy, and the number of displacement events. These correlation analyses were interpreted as exploratory given the observational design and the likelihood of measurement constraints and incomplete capture of pregnancies not reaching facility care.

When documentation was incomplete, variables were coded according to the available categories (e.g., caesarean section indication “not identified”). Denominators are therefore variable‐specific and reflect the number of encounters/newborn records that had available documentation.

## 3 RESULTS

Among 686 women (age range 15–47 years; median 28 [IQR 23–33]), indicators of deprivation and conflict exposure were highly prevalent (Table 1). Most women reported ≤2 meals/day (92.9%) and were currently displaced (78.7%, median 4 displacement events [IQR 2–5]), and many lived in a tent (54.8%). Exposure to nearby Israeli bombing in the prior 24 h was reported by 57.0%, exposure to smoke by 98.0%, and heavy lifting by 74.6% (Table 2).

**TABLE 1:** Sociodemographic, clinical, and obstetric histories of the mothers.

Variable	Median (IQR) or *n* (%)
Age, range (15–47)	28 (33–23)
Daily meals	
2 or fewer	637 (92.9%)
3 or more	49 (7.1%)
Gravidity	
1–4	453 (66%)
5–9	208 (30.3%)
10–15	25 (3.6%)
Term	
0–3	455 (66.3%)
4–7	207 (30.2%)
8–11	24 (3.5%)
Preterm	
0–2	672 (98%)
3–6	14 (2%)
Abortion	
0–3	665 (96.9%)
4–10	21 (3.1%)
Living	
0–3	430 (62.7%)
4–7	233 (34%)
8–11	23 (3.4%)
Gestational age	
≥37 weeks	554 (80.8%)
34–36	64 (9.3%)
32–33	13 (1.9%)
28–31	14 (2%)
24–27	5 (0.7%)
<23 weeks	36 (5.2%)
Hemoglobin levels	
≥11 g/dL	194 (28.3%)
<11 g/dL	492 (71.7%)
Medical history	
Depression	1 (0.1%)
HBV	3 (0.4%)
Epilepsy	1 (0.1%)
ITP	1 (0.1%)
Thrombophilia	6 (0.9%)
Arrythmia	2 (0.3%)
Hypothyroidism	4 (0.6%)
Hyperthyroidism	1 (0.1%)
Rheumatoid arthritis	1 (0.1%)
Asthma	7 (1%)
Thalassemia carrier	9 (1.3%)
Chronic hypertension	16 (2.3%)
Chronic diabetes mellitus	6 (0.9%)
Pyelonephritis	2 (0.3%)

Abbreviations: HPV, human papillomavirus; ITP, immune thrombocytopenia.

**TABLE 2:** Conflict‐related exposures and living conditions.

Variable	Median (IQR) or *n* (%)
Conflict‐related injury before pregnancy	73 (10.6%)
Disability due to conflict‐related injury before current pregnancy	8/73 (11%)
Nearby bombing in the last 24 h	391 (57%)
Forced displacement in the last 24 h	85 (12.4%)
Currently displaced	540 (78.7%)
Number of displacements, range (0–20)	4 (5–2)
Conflict‐related trauma in the current pregnancy	62 (9%)
Surgical intervention for conflict‐related trauma	17 (2.5%)
Living in tent	376 (54.8%)
Exposure to smoke	672 (98%)
Lifting heavy object(s)	512 (74.6%)
Children killed	26 (3.8%)
Death of spouse due to conflict‐related injury	26 (3.8%)
Conflict‐related injury in spouse	179 (26.1%)
Disability in spouse due to conflict‐related trauma (*n =* 179)	70 (39.1%)

Anemia (Hb <11 g/dL) occurred in 71.7%, and reported access to prenatal care was low (32.1%) (Table 3). Delivery management included normal vaginal delivery in 57.4% and caesarean section in 37.6% (most commonly due to prior caesarean). Postpartum ICU admission occurred in 1.0% (*n =* 7), and two deaths were recorded among ICU admissions.

**TABLE 3:** Characteristics and complications of the current pregnancy.

Variable	Median (IQR) or *n* (%)
Management of the current pregnancy	
NVD	394 (57.4%)
C‐section	258 (37.6%)
D&C	27 (3.9%)
Others^a^	7 (1%)
CS indication	
Previous CS	138/258 (53.4%)
Breech	33/258 (12.8%)
Fetal distress	28/258 (10.9%)
Twins	6/258/(2.3%)
Others^b^	34/25 (13.2%)
Not identified	19/258 (7.4%)
Postpartum ICU admission	7 (1%)
ICU admission indications	
Shock	5/7 (71.4%)
Eclampsia	2/7 (28.6%)
Post ICU status	
Alive	5/7 (71.4%)
Dead	2/7 (28.6%)
Prenatal care accessibility and availability	220 (32.1%)
Unplanned pregnancy	361 (52.6%)
Seeking for contraceptive in unplanned pregnancy (*n* = 361)	150 (41.6%)
Accessibility and availability of contraceptive method in unplanned pregnancy (*n* = 150)	43 (28.7%)
Pregnancy outcome	
Single	646/665 (97.1%)
Multiple	19/665 (2.9%)
PROM	34 (5%)
Placenta abruptio	7 (1%)
Placenta Previa	6 (0.9%)
Postdate pregnancy	16 (2.3%)
Pregnancy induced hypertension	
Gestational hypertension	25 (3.6%)
Pre‐eclampsia	11 (1.6%)
Eclampsia	2 (0.3%)
GDM	12 (1.7%)
Ectopic pregnancy	2 (0.3%)
IUGR	1 (0.1%)
Breech presentation	36 (5.2%)

Abbreviations: GDM, gestational diabetes mellitus; IUGR, intrauterine growth restriction; PROM, prelabor rupture of membrane.

^a^ Methotrexate, misoprostol, surgical salpingectomy.

^b^ Eclampsia, precious baby, failed induction.

Among 665 newborn records, median birthweight was 3000 g (IQR 2600–3300) and NICU admission occurred in 12.9% (Table 4). The last documented Apgar score was 10 in 43.0% and 0 in 9.5%. Discharge status was recorded as alive in 98.6%. A correlation analysis was also conducted between newborn weights and the number of meals, hemoglobin level, whether it was a planned pregnancy, and the number of displacement events (Table 5). These figures are not necessarily extreme by themselves; rather, their significance lies in their co‐occurrence with the conditions of famine, multiple displacement events, and exposure to toxic chemicals in Gaza.^43^

**TABLE 4:** Demographic and clinical characteristics of neonates.

Variable	Median (IQR) or *n* (%)
Newborn gender (*n =* 665)	
Female	307 (46.2%)
Male	358 (53.8%)
Abortions, ectopic pregnancy, or IUFD	40 (5.8%)
Newborn weight, range (650–4500)	3000 (3300–2600)
The last documented Apgar score	
0	63 (9.5%)
1	1 (0.1%)
2	2 (0.3%)
4	4 (0.6%)
5	12 (1.7%)
6	18 (2.6%)
7	14 (2%)
8	106 (15.5%)
9	180 (26.2%)
10	286 (43%)
Newborn status (*n* = 665)	
Alive	656 (98.6%)
Dead	9 (1.4%)
Neonatal admission to ICU	86 (12.9%)

**TABLE 5:** Correlation analysis between newborn weights and other items.

Items	Items	Correlation coefficient	*P*‐value
Number of meals	Newborn weights	0.04	0.25
Hemoglobin levels		0.05	0.18
Planned pregnancy		−0.17	0.66
Number of displacements		−0.009	0.812

## 4 DISCUSSION

Pregnancy and birth itself have become life‐threatening events in Gaza amid the ongoing Israeli‐led genocide.^10^, ^21^, ^44^ We retrospectively and prospectively collected data on 686 women from the Al‐Helou Maternity Hospital (and some from the Al‐Shifa Medical Complex) in the Gaza governorate in Palestine with the aim of systematically documenting clinical outcomes alongside conflict‐related exposures and living conditions. Since the resumption of large‐scale Israeli airstrikes and violation of the ceasefire agreement in March 2025, the picture that emerges is of women attempting to carry pregnancies to term while hungry, displaced, and inhaling smoke, conditions that predictably harm mothers and newborns.^6^

### 4.1 Maternal health outcomes

This study provides an empirical record of the maternal and neonatal health related consequences that have been caused by the Israeli military assault on Gaza. Our findings reflect the collapse of many vital maternal and neonatal health indicators primarily driven by the biosphere surrounded armed conflict and genocide, a state in which the environmental, nutritional, and psychological factors affecting daily life can become incongruent with healthy pregnancy and gestation. In our cohort, 78.7% women were forcibly displaced at the time of their clinical encounter, with a median of four displacement events since October 2023; 54.8% of these women resided in tents, where conditions are notably unhygienic with the lack of access to clean sanitation and toilets.^45^

Such physical difficulties have been compounded by the acute effects of ongoing bombardment, with 57% of participants reporting that they experienced bombing nearby within the last 24 h prior to admission. These indiscriminate attacks against civilians can produce sustained psychological trauma that clinicians have identified as being potential drivers for pregnancy‐related complications and stress‐induced miscarriages.^3^, ^21^, ^46^, ^47^, ^48^ Chronic exposure to bombardment, repeated events of forced displacement, and even the fear of death itself can generate population‐wide states of anxiety, be it civilians, frontline healthcare workers, or other affected populations, that can, in turn, lead to prolonged cortisol elevation.^22^, ^23^, ^49^, ^50^, ^51^, ^52^, ^53^, ^54^ This stress is also amplified by personal loss, with 3.8% of women reporting having a child killed during the current armed hostilities, and a similar proportion reported the death of a spouse. Approximately one‐quarter of pregnant women's spouses had suffered conflict‐related injuries, with 39% of these injuries resulting in disability.

Nearly one in five pregnancies in this study ended preterm (<37 weeks), approximately 7% resulted in a medically necessary abortion, ectopic pregnancy, intrauterine fetal demise, or with the neonate dying shortly after birth. Premature rupture of membranes and placental abruption was also present in this dataset. Some complications were directly consistent with conflict‐related trauma and widespread resource shortages.

The conditions of life in Gaza are in stark contrast to what is often recommended for a healthy pregnancy and birthing experience.^55^ For example, approximately 75% of women reported having to lift heavy objects, in large part as a survival necessity given the displacement of tents, shelter, and carrying of food aid.^56^ Simultaneously, the environmental degradation present in Gaza has somewhat fundamentally altered the biological baseline of gestation. Nearly all women (98.0%) were exposed to fire and smoke inhalation chronically (as a consequence of the lack of cooking gas and heavy bombardment), and 71.7% presented with clinical anemia (Hb <11 g/dL), which, in turn, can create a state of sustained maternal–fetal hypoxia. Overcrowding in tents and refugee camps, damaged sewage infrastructure, and the absence of hygiene supplies can exacerbate the spread of infections, including urinary tract infections and hepatitis A, both of which risk increasing obstetric complications and morbidity.^2^, ^57^, ^58^ Exposure to airborne pollution and combustion products, much of which is generated after Israeli bombardment, has been linked to increased rates of miscarriages and spontaneous abortions.^59^

### 4.2 Neonatal outcomes

Newborns, particularly those born preterm, frequently present with respiratory distress, which is compounded by the scarcity of surfactant, oxygen supplies, and functional neonatal equipment. The Apgar score is a measure of a newborn's immediate health at birth and can indirectly reflect maternal health and delivery conditions.^60^ A population‐wide depletion of fetal metabolic reserve is somewhat reflected in the high burden of depressed Apgar scores in our cohort, with only 43% of newborns scoring 10, potentially indicating a high prevalence of stillbirths and neonatal deaths in an environment where timely resuscitation is often impossible. Factors such as maternal distress, certain medication usage, age, body mass index, and pregnancy‐related complications can influence a neonate's Apgar score. A low Apgar score, especially at the 1‐ or 5‐min mark, can indicate a poor prognosis for survival, as it is potentially reflective of an infant's struggle to breath, maintain circulation, or regulate tone.^61^

The Israeli military‐led blockade of Gaza has limited not only necessary medical and surgical supplies including anesthetics, incubators, and ventilators but also targeted sources of electrical supply, water desalination plants, and fuel reserves that can be necessary for hospitals, generators, and incubators to function.^62^, ^63^, ^64^ Timely resuscitation with oxygen is often rationed, as oxygen tanks are frequently brought and refilled from neighboring hospitals with staff often traversing areas of active Israeli military activity to obtain minimal amounts of oxygen. This shortage of incubators and ventilators has forced some babies to share oxygen masks to merely stay alive, with the Israeli military often denying hospital personnel from recovering vital equipment such as incubators stranded inside hospitals that have been encircled, sieged, destroyed, or left in “red zones” marked for killing.^65^

Maternal nutrition is imperative for the health and well‐being of both mother and fetus. Pregnancy is a physiologically demanding process that can drain mothers of their metabolic reserves, requiring them to nourish themselves well beyond their regular baseline to maintain health. Israel has imposed a starvation strategy in Gaza, with the IPC declaring famine in the Gaza governorate during the same period that this study was conducted.^43^, ^66^ Malnutrition had already reached record‐high levels in Gaza amid the genocide, with 15.8% of children screened by early August 2025 reporting wasting.^67^, ^68^ Such caloric and nutritional deficient in expecting mothers can lead to miscarriages, premature labor, and stillbirths.^6^, ^69^, ^70^ In our study, approximately 93% of women reported eating two or fewer meals per day, which, combined with other factors regarding the type of foods allowed into Gaza and portion sizes, suggests a starvation diet.^71^

### 4.3 Implications of findings and limitations

A simple correlation analysis did not detect statistically significant linear associations between birthweight and number of meals, hemoglobin, pregnancy planning, or number of displacements. This absence of signal should not be misread as absence of effect. The combination of constrained variance in birthweight, potential misclassification of exposures, survivorship bias (pregnancies ending before hospital contact), and unmeasured confounding (infection, micronutrient deficits, smoking fuel type, and interval from last meal) can easily attenuate linear correlations. Part of the interpretation is limited by potential exposure misclassification (self‐report), restricted variance in some exposures (e.g., near‐universal exposure to smoke), survivorship bias (pregnancies not reaching facility care), and unmeasured confounding (e.g., infection, micronutrient deficiency, gestational age at measurement, and timing/quantity of food intake). The descriptive data themselves regarding limited food consumption, inhalation of smoke, forced displacement, and being subject to bombing are clear markers of the risk environment. Some of these figures are not extreme by themselves; rather, their significance lies in their co‐occurrence with the conditions of famine, multiple displacement events, and exposure to toxic chemicals in Gaza.^43^

Before the current genocide, when there was frequent Israeli military assaults on the Gaza strip and already decades of military occupation, many of the obstetric and newborn indicators paralleled what is seen in other resource‐constrained settings. For example, in the Gaza Infant Study (*n =* 511, recruited through 10 governmental primary health‐care centers across all five governorates, followed from the second trimester through to 12 months postpartum), exposure to conflict‐related trauma during the 2008–2009 Israeli military assault was associated with higher prenatal and postpartum post‐traumatic stress disorder/depressive/anxiety symptoms and a small increase in pregnancy complications.^72^ Nevertheless, it was not directly associated with prematurity, low birthweight, or newborn health risks, and many of the adverse effects on infant sensorimotor/language development at 12 months were often mediated through maternal mental health rather than being as a consequence of newborn complications.^72^ In that cohort, reported pregnancy complications such as miscarriage risk or ultrasound abnormalities were typically in the approximately 5–8% range. High blood pressure was approximately 2.5%, prematurity was approximately 5%, and approximately 14% of newborns required immediate control or intensive care after birth.^72^ Such data from before 2023 showcases that the current patterns we document, of pervasive hunger, repeated displacement, continued exposure to smoke, and severe constraints on obstetric/neonatal care, represent more than just the effects of a routine war, but rather the impact of health harms compounded due to physiological and health system changes.

What can be done is specific, feasible, and urgent. First, secure protected, uninterrupted humanitarian corridors to deliver essential equipment and medicines for obstetric and neonatal care such as magnesium sulphate, antihypertensives, oxytocin, tranexamic acid, antibiotics, Rh immunoglobulin, neonatal resuscitation equipment, reliable oxygen and fuel for theaters, blood banks, and NICUs. Second, steps can be undertaken to re‐establish antenatal care through mobile midwife‐led teams embedded in shelters and camps, offering blood pressure and urine protein screening, iron–folate supplementation, simplified gestational diabetes screening, and tetanus immunization. Every contact should include mid‐upper‐arm circumference and screening for hunger.^67^ Third, referrals should be processed through the World Health Organization for cross‐border evacuations when necessary and desired by patients for high‐risk pregnancies (e.g., severe pre‐eclampsia, placenta previa/abruption, labor after prior caesarean section, and extreme prematurity). Healthcare workers can pressure their governments to facilitate unimpeded access to such corridors and their hospitals to accept such cases. As always, demanding protection and access for civilians and health facilities in line with international humanitarian law is imperative: be it maternity wards, ambulances, staff housing, or marked evacuation routes.

The data from the Al‐Helou Maternity Hospital does not sensationalize. It simply presents what this genocide has made routine: hungry, smoke‐exposed, repeatedly displaced women laboring in hospitals that are themselves under threat. The result is a caesarean‐heavy service, limited neonatal resuscitation, and avoidable losses. Maternal and newborn outcomes are not a post‐conflict concern—they are the present‐tense barometer of humanitarian performance. Every uninterrupted meal, every functioning antenatal care contact, every protected ambulance corridor can move these numbers. The question is whether the international system will act quickly enough for the next woman who arrives at Al‐Helou or hospitals in Gaza in labor, or if they will even be able to access such a facility.

## AUTHOR CONTRIBUTIONS

Shaymaa Abuhaiba: Conceptualization, Data curation, Investigation, Project administration, Writing–review & editing. Younis Elijla: Formal analysis, Writing–review & editing. Aya El‐Dadah: Investigation, Writing–review & editing. Othman Nassar: Investigation, Writing–review & editing. Meran Abu‐Sultan: Investigation, Writing–review & editing. Maysaa Almasry: Investigation, Writing–review & editing. Hebatallah Alkhatib: Investigation, Writing–review & editing. Mahmoud Alsady: Investigation, Writing–review & editing. Amir Mahdi Ghafarian: Writing–original draft, Writing–review & editing. Muath Alsarafandi: Formal analysis, Writing–review & editing. Lina Abumughessib: Writing–review & editing. Arham Ali: Writing–review & editing. Yassar Arain: Writing–review & editing. Abeerah Muhammad: Writing–review & editing. Belal Aldabbour: Writing–review & editing. Shayan Ali: Writing–review & editing. Bilal Irfan: Conceptualization, Data curation, Project administration, Supervision, Writing–original draft, Writing–review & editing.

## CONFLICT OF INTEREST STATEMENT

The authors declare no competing interests regarding this article.

## Data Availability

Research data is available upon reasonable request to the corresponding author.
